# Effects of Contact Sports on Temporomandibular Disorders: An Observational Study

**DOI:** 10.3390/dj10100180

**Published:** 2022-09-27

**Authors:** Vito Crincoli, Corrado De Biase, Angela Pia Cazzolla, Alessandra Campobasso, Mario Dioguardi, Maria Grazia Piancino, Luigi Mattia, Domenico Ribatti, Mariasevera Di Comite

**Affiliations:** 1 Interdisciplinary Department of Medicine, University of Bari Aldo Moro, Piazza G. Cesare 11, 70124 Bari, Italy; 2 Department of Surgical Sciences, I.R.C. Dental School, University of Turin, Via Nizza 230, 10126 Turin, Italy; 3 Department of Clinical and Experimental Medicine, University of Foggia, Clinica Odontoiatrica, Via Rovelli 50, 71122 Foggia, Italy; 4 Department of Dental Sciences, University of Bologna, Via San Vitale 59, 40125 Bologna, Italy; 5 Department of Basic Medical Sciences, Neurosciences and Sensory Organs, Division of Complex Operating Unit of Dentistry, University of Bari Aldo Moro, Piazza G. Cesare 11, 70124 Bari, Italy

**Keywords:** contact sports, temporomandibular disorders, mandibular movements, TMJ sounds, orofacial pain

## Abstract

The study investigated the prevalence of temporomandibular disorders in 100 competitive athletes in contact sports, equally grouped by the practiced game: Soccer (SoG), Rugby (RG), American Football (AFG), Boxing (BoG), Basketball (BaG), compared to a randomly control group of 20 non-athletes (CG). Symptoms and signs were examined according to the standardized Diagnostic Criteria for Temporomandibular Disorders through a questionnaire and clinical evaluation. Arthralgia showed significant differences between RG and CG and between AFG and CG (*p* < 0.05). Study groups reported masticatory muscle pain during function, neck and shoulder pain more frequently than CG, except for BoG. Closing click was significantly more present in study groups than CG, while crepitation was significantly higher only in RG and AFG. The deviation was wider in SoG, RG and AFG compared to CG (*p* < 0.05). Tukey’s multiple comparisons test showed a statistically significant reduction in right laterotrusion in RG vs. CG (*p* < 0.05); the comparison showed a decrease in right laterotrusion in RG vs. SoG and BoG (*p* < 0.05), a decrease in endfeel in RG vs. CG, BaG and AFG (*p* < 0.05). The data seem to support a relationship between the prevalence of TMD symptoms and signs in competitive athletes in contact sports, especially in RG and AFG compared to CG.

## 1. Introduction

Temporomandibular disorders (TMD) are a group of craniofacial disorders involving the temporomandibular joint (TMJ), the masticatory muscles and the musculoskeletal structures of the head and neck [1]. The TMD are frequently characterized by orofacial pain, limited or asymmetric mandibular movements and TMJ sounds [2]. Other symptoms, such as tinnitus, neck pain and headache, may also occur [1]. Symptoms can range from a self-limiting mild discomfort to chronic debilitating pain, with psychological implications [3]. Among adults, the prevalence of TMD ranges from 1% to 75% for objective signs and 5% to 33% for subjective symptoms [4]. TMD’s etiology is multifactorial and often influenced by age, gender, hormonal imbalances, traumatic injuries, stress and other systemic diseases [3,5,6,7]. Age plays an etiological role in TMD: the prevalence of TMD is usually more common in young adults and the range from 20 to 40 years of age coincides with the peak of the incidence of TMD, as reported by previous studies [3,4,5,6,8]. Gender is also a significant risk factor for the development of TMD: the female gender has a two up to seven times higher risk of developing TMD than the male gender [9]. The increased prevalence of TMD in women is probably due to differences in estrogen levels and corresponding signaling mechanisms. Indeed, the literature has reported that the fluctuations in estrogen and corresponding changes in other sexual hormones (such as progesterone) could contribute to the pathogenesis of TMD [10].

This could explain why the onset of symptoms usually occurs after puberty and is reduced in old age [11]. In addition, trauma seems to contribute to the onset of TMD and can be classified as macrotrauma and microtrauma [12]. Microtrauma is due to internal factors, such as parafunctional habit (bruxism), malocclusion [6] or estrogens that can lead to TMJ degeneration, damaging the condylar fibrocartilage, the disk and the entire TMJ structure [9,10]. Macrotrauma is due to injury from outside and it can be further classified as direct and indirect. In the direct trauma there is a direct injury to the mandible, while in the indirect trauma there is no direct contact between the impact and the TMJ [6]. Both can induce a loss of the structural integrity of TMJ or can damage the ligaments and the soft tissues through traumatic stretching, with resulting disk displacement or TMJ effusion [12]. Even if a female predominance of TMD has been reported mostly due to microtrauma, a male predominance of TMD was reported among patients with macrotraumatic etiology [13]. The causes of traumatic injuries are several, including road accidents, third molar extraction, physical violence and contact sports [6]. Contact sports are reported to be an etiological factor in the development of TMD because they are associated with a higher risk of injury of the orofacial district, due to the intentional or accidental physical contact among players [14,15,16,17,18]. As well as macrotrauma, the psychological impairment of competitive athletic activity can also contribute to the development of TMD. The competitive athletes are exposed to greater stress compared to non-athletes, due to the high training intensity [16] or to the psychological pressure of competitions and of increasing training effort [19,20]. Although literature reports as a general trend that athletes suffer from TMD more frequently than non-athletes [21], the available evidence of the effects of competitive contact sports on TMD is currently scarce. The aim of the present study was to investigate the prevalence of TMD in competitive athletes in the main contact sports compared to a control group of non-athletes. The null hypothesis of the study was that no differences occurred between groups.

## 2. Materials and Methods

This clinical observational study was conducted from March 2021 to November 2021 at the School of Dentistry of the University of Foggia, Italy, in accordance with the provisions of the Declaration of Helsinki. Before entering the study, all subjects signed informed consent. Ethical approval was obtained by the Research Ethics Committee of the “Ospedali Riuniti” of Foggia: N°145/CE/2021.

One hundred male patients were recruited and equally divided into five study groups (20 patients for each group) based on the sport practiced: Soccer group (SoG), Rugby group (RG), American Football group (AFG), Boxing group (BoG), Basketball group (BaG). The inclusion criteria were: competitive athletes affiliated with sports federations and playing in regional championships, with a sports seniority of 15 ± 3 years; general good health; patients’ age ranging between 18 and 40 years; Caucasian ethnic origin. Exclusion criteria were: amateur athletes; head, oral and neck neoplasia; past or present chemotherapy and radiotherapy; neurological disorders; previous maxillofacial treatment. A control group (CG) of 20 non-athletes, matched by sex (20 men) and age with the study groups, was randomly chosen from those who underwent a routine oral check at the School of Dentistry of Foggia. Before starting the study, the randomization list was automatically generated (Microsoft Excel, ver. 2207; Microsoft Corporation, Redmond, WA, USA). TMD signs and symptoms were evaluated following the standardized Diagnostic Criteria for Temporomandibular Disorders (DC/TMD). A single blinded experienced operator conducted the procedure, in order to reduce the bias. This practitioner, one of the co-authors, is a specialist in Orthodontics and the University Professor of Gnathology. He assessed TMD and orofacial manifestations through an anamnestic questionnaire and a clinical examination.

Symptoms and signs of the study groups were compared to those present in the CG.

### 2.1. Patient History

The history questionnaire [Supplementary materials] was used to assess: (1) Clinical anamnesis; (2) TMD symptoms.

#### 2.1.1. Clinical Anamnesis

The anamnesis was performed for each athlete, collecting any comorbidities, drugs, supplements and reported trauma. The occlusal data, as well as facial asymmetry, presence of scars, any previous orthodontic treatment and the possible use of a mouthguard were also recorded.

#### 2.1.2. TMD Symptoms (TMDs)

Patients were asked to categorically report the presence or absence of the following TMD symptoms: tension or pain of the masticatory muscles, both at rest and during jaw movements; tension or pain of the shoulder and neck muscles; painful TMJ (arthralgia); difficult mouth opening; headaches located in the temporal or preauricular area and/or in the masseter region; tinnitus and locks [22,23,24].

To prove the TMD etiology in the onset of headaches, the patient should report at least two of the following criteria in addition to clinical evidence of TMD:

(i) the onset of headaches coincides temporally with the onset of TMDs;
(ii) the headache is evoked by palpation of the temporalis muscle or by passive movements of the jaws;
(iii) the onset or disappearance of the headache and TMD coincide in time with each other [25].

### 2.2. Clinical Examination

The clinical examination was performed to assess: (1) the presence of myofascial pain; (2) the presence of TMD signs; (3) the mandibular kinematics, with any restrictions on mandibular movements.

#### 2.2.1. Myofascial Pain (MP)

The manual palpation of the neck and masticatory muscles was performed on the right and left sides separately, applying soft firm pressure on the muscles. If they are inflamed or contracted, pain during pressure occurs [26]. The following muscles were palpated extra orally: anterior, middle and posterior temporalis, masseter, internal pterygoid, digastric, sternocleidomastoid and trapezius. The external pterygoid muscles were palpated intraorally. Flat palpation (e.g., on temporalis muscle) or pincher palpation (e.g., on masseter, trapezius, and sternocleidomastoid muscles) were carried out. The digital pressure was standardized using an algometer (model DDK-20; KRATOS Equipamentos Industriais) to calibrate the finger pressure [27]. The palpation was carried out with a pressure of 1.0 kgf to the extraoral sites and approximately 0.5 kgf to the intraoral and articular sites [28], as recommended by the RDC/TMD [29]. These data were categorically collected (presence or absence of muscular pain).

#### 2.2.2. TMJ Signs

(a) TMJ sounds (TMJs): they refer to the condylar sound during mandibular movement and are perceived by digital palpation of the lateral TMJ area for each side [30]. They can be classified into:
  (i) clicking, defined as a single, sharp and net click sound [31],
  (ii) snapping, characterized by a louder and sharper sound as a “pop noise” [32],
  (iii) crepitation, defined by multiple gravel-like sounds [33,34];
(b) Parafunctional signs: bruxism is a repetitive jaw muscle activity characterized by grinding or clenching of the teeth during sleep or while awake [34]. It can induce pathological changes, such as the onset of muscular (myalgia) and joint (arthralgia) pain and the appearance of oral signs, such as dental wears, irregular lingual edges and buccal occlusal line [3,4,5,6,8,9,10,11,12,13,14,15,16,17,18,19,20,21,22,23,25,26,27,28,29,30,31,35]. The diagnosis of bruxism was carried out through non-instrumental assessment based on self-reported information (medical history, questionnaires) and clinical examination [36]. Patients were asked whether they had the habit of grinding or clenching their teeth during the day or whether they had been reported to have this habit during sleep. Self-reported assessment of sleep or awake bruxism continues to be the primary tool in bruxism research and clinical practice [36], so a positive self-report was considered suggestive of possible bruxism;
(c) Mandibular opening alterations: in a healthy condition, the trajectory of mouth opening is straight. The mandibular opening alterations are:
  (i) deviation: the shift of jaw to one side from midline with turn back to the midline at the opening of the mouth;
  (ii) deflection: shift of jaw to one side without turn back to the midline at the opening of the mouth.

#### 2.2.3. Mandibular Kinematics (MK) and Restriction of Movements (RM):

(i) Reduced opening: the mouth opening relates to the distance between the upper and lower incisal edges which physiologically corresponds to a value between 53 and 58 millimeters. A reduced opening occurs when this value is lower than 40 mm [4];
(ii) Endfeel distance: this parameter is assessed by applying a strong force with the thumb and index fingers between the anterior teeth at the maximal active mouth opening to passively increase the incisal distance beyond the maximal mouth opening. Both the active and the passive openings were measured through a caliper. Normally, the endfeel value should be 2 mm, due to the physiological stretching of the ligaments (joint play) [31]. The endfeel is positive when the value exceeds 2 mm, indicating a muscular contracture that is evaluated in numerical terms [37];
(iii) Lateral excursion (right and left): a reduced lateral excursion was recorded when the distance from upper to lower midline was <8 mm [32];
(iv) Mandibular protrusion: under healthy conditions, the mean protrusive values range between 7 and 10 mm. A reduced mandibular protrusion was recorded when <7 mm [32,38].

## 3. Statistical Analysis

A sample size (α = 0.05; β = 0.2; power = 80%) for two independent study groups and dichotomous endpoint was calculated considering the “Closing click” variable. The anticipated incidence was supposed to be 41% and 5% for athletes and control groups, respectively. The calculation required 20 patients per group for the study. For Effect size analysis, Relative Risk, Attributable risk, Odds ratio, sensitivity and specificity were performed by Koopman asymptotic score, Newcombe/Wilson score, the Baptista–Pike method and the Wilson–Brown method, respectively.

Dichotomous data were expressed as numbers and percentages. The comparisons between study groups and control patients were performed using the Chi-squared **^¥^** test and, if not applicable because the expected value of a cell was <5, the Fisher exact test **^‡^**. Quantitative data were presented as mean and Standard Deviation (SD) and the comparisons among groups were valued using ordinary one-way ANOVA and post hoc Tukey’s multiple comparisons tests. In all comparisons, a *p* value <0.05 was considered statistically significant. Statistical analyses and graphs were performed using Prism (GraphPad software, version 9.3.1 San Diego, CA, USA). Asterisks point out statistically significant values in the tables and graphs.

## 4. Results

Data about mean ages (±SD) per group are shown in Table 1.

### 4.1. Comorbidity and Drugs Taken

Significant differences were found between SoG and CG, because soccer players did not suffer from systemic pathologies (*p* = 0.00) and did not take medications (*p* = 0.02) compared to controls.

Overlapping differences were also found between RG and CG: rugby athletes did not suffer from systemic pathologies (*p* = 0.00) and did not take any medications (*p* = 0.02), although, conversely, these athletes reported a significant increasing amount of trauma (*p* = 0.03) compared to controls.

The AFG did not take medications (*p* = 0.02) compared to controls and showed a statistically significant scar reduction (*p* = 0.02) and a statistically greater number of previous trauma (*p* < 0.01)

No statistically significant differences were found between the BoG and CG and between BaG and CG, except for reported traumas that were more frequent in basketball players than in controls (*p* < 0.01). Comorbidity and drugs data are reported in Table 2.

### 4.2. Occlusion and Use of Mouthguard

About these parameters, no statistically significant differences were found between soccer players and controls, except for a reduction in orthodontic treatments (*p* < 0.01).

In the RG, a statistically greater number of players had cross-bites (*p* = 0.02) and used mouthguards (*p* = 0.00), while a statistically significant reduction in orthodontic treatments (*p* = 0.00) was present compared to the CG. No other differences were found.

Orthodontic treatments (*p* = 0.00) and midline deviation (*p* = 0.01) were significantly more frequent in the CG than in AFG, while the use of mouthguards (*p* = 0.00) was far more common in AF players.

Mouthguards were significantly more frequent in the BoG than in the controls (*p* = 0.00).

The alterations of the midline (35% BaG vs. 75% CG, *p* = 0.01) and the use of mouthguards was more frequent in the controls (0% BG vs. 25% CG, *p* = 0.00) compared to basketball players, with a statistically significant difference. Occlusion data are reported in Table 3.

### 4.3. TMD Symptoms

Regarding the TMD symptoms, 40% of soccer players complained of muscle pain during function compared to 5% of controls (*p* < 0.01) and, in addition, 65% of SoG had neck and shoulder pain compared to 25% of controls (*p* = 0.01): both these differences are statistically significant.

In the RG, 40% of the rugby players complained of muscle pain when chewing, compared to 5% of controls (*p* < 0.01). In addition, 65% of rugby players reported neck and shoulder pain (*p* = 0.01), 40% TMJ arthralgia (*p* = 0.03) and 25% difficulty opening their mouth (*p* = 0.02). These differences were all statistically significant compared to the CG.

AF players most frequently reported TMD symptoms. In detail, 55% of athletes complained of muscle pain when chewing, compared to 1% of controls (*p* = 0.00). In addition, 70% of footballers had neck and shoulder pain (*p* = 0.00), 40% suffered with TMJ arthralgia (*p* = 0.03) and 65% reported temple headaches (*p* = 0.03) compared to controls, with a statistical significance.

TMD symptoms showed no differences between the boxing group and controls. Only tinnitus was significantly less frequent in BoG than in the CG (*p* < 0.05).

Basketball players experienced significant myofascial pain during chewing more frequently than controls (*p* = 0.02). No more statistically significant differences were found between BaG athletes and controls. TMD symptoms data are reported in Table 4.

### 4.4. Myofascial Pain

The soccer players tended to suffer from muscle aches during palpation, although statistically significant differences with the CG were found only for the anterior temporal muscle (45% SoG vs. 10% CG, *p* = 0.01); on the contrary, the digastric muscle was less painful on palpation in the SoG than the CG (10% SoG vs. 60% CG, *p* = 0.00).

No differences in myofascial pain were found between the rugby players and controls. Among AF athletes, no differences between the study group and controls were found regarding muscular symptoms except for the anterior temporal muscle (*p* = 0.01), which was more frequently painful on palpation in the AFG.

Myofascial pain tended to be more frequent in the CG than in the boxing group, even if statistically significant differences were found only for the sternocleidomastoid (*p* = 0.04), superficial masseter (*p* < 0.05), medial pterygoid (*p* < 0.02) and lateral pterygoid (*p* = 0.00) muscles.

Among the muscles evaluated, the digastric (*p* = 0.03) and lateral pterygoid (*p* = 0.02) were less painful during palpation in the basketball players than in controls and this comparison was statistically significant. Muscular symptoms data are reported in Table 5.

### 4.5. TMJ Sounds

About joint sounds, the closing click was significantly more present in soccer players than in the control group (60% SoG vs. 1% CG, *p* = 0.00). Other joint sounds were indistinctly present in both SoG and CG (*p* > 0.05).

Rugby players showed an increase in TMJ sounds than controls: in detail, the evaluations of the closing click (*p* = 0.00) and the crepitation (*p* = 0.02) were statistically significant.

The TMJ sounds’ evaluation revealed a significant greater positivity at the closing click (*p* = 0.00) and crepitation (*p* < 0.05) in the AFG compared to CG.

The closing click was also more frequent in the BoG (*p* = 0.02) than in controls. This difference was statistically significant such as in the comparison between BaG and CG, in which the closing click was more significantly frequent in basketball players (*p* = 0.00) than in controls. Joint sound data are reported in Table 6.

### 4.6. Parafunctional Signs

Regarding bruxism and its oral signs, the statistical analyses showed no significant difference between the SoG and CG, RG and CG and AFG and CG.

In the comparison between BoG and CG, the statistical analysis did not show any significant difference between the investigated groups except for the buccal occlusal line (*p* = 0.00), which appeared less frequently in the boxing group.

Some parafunctional signs, such as the indentation of lateral edges of the tongue (*p* = 0.01) and the buccal occlusal line (*p* = 0.00), were statistically less common in the basketball players than in controls. Parafunctional signs’ data are reported in Table 7.

### 4.7. Mandibular Opening Alterations

Regarding qualitative alterations of the buccal opening, the mandibular deviation was statistically most recurrent in the SoG (50%) than in the CG (20%), (*p* < 0.05).

Fifty-five percent of the rugby athletes reported a deviation in the mouth opening compared to 20% of controls (*p* = 0.02).

Among the AF players, 55% of the athletes compared to 20% of controls (*p* = 0.02) reported a deviation in the mouth opening.

No statistically significant differences were found in mandibular deviation and deflection, between the BoG and CG and between BaG and CG. The opening alteration data are reported in Table 8.

### 4.8. Mandibular Kinematics

The mean values and ± standard deviation (SD) of active opening measured in athletes of different groups and the controls revealed no statistically significant differences.

ANOVA and Tukey’s multiple comparison tests (Table 9) showed that the opening did not vary among the sports groups (F = 1.47, *p* = 0.21). Regarding endfeel, the ANOVA test revealed statistically significant differences among the different groups (F = 3.52, *p* = 0.01). In detail, Tukey’s multiple comparisons tests showed a statistically significant decrease in endfeel in the Rugby Group (RG) vs. controls (* adjusted *p* value = 0.03), vs. BaG (*** adjusted *p* value = 0.01) and vs. AFG (* adjusted *p* value = 0.03).

Right laterotrusion showed statistical differences among the groups evaluated (F = 4.62, *p* = 0.00). In detail, Tukey’s multiple comparisons test highlighted the presence of a statistically significant reduction in right laterotrusion in the Rugby group vs. controls (*** adjusted *p* = 0.00); the comparison among the groups of athletes also showed a decrease in right laterotrusion in RG vs. SoG group (** adjusted *p* < 0.02) and BoG (*** adjusted *p* =0.01). Finally, ANOVA and Tukey’s multiple comparison tests did not reveal any significant differences for left laterotrusion (F = 0.84, *p* = 0.52) and protrusion (F = 1.54, *p* = 0.18) measured in different groups (Table 10).

## 5. Discussion

Currently, very few studies have focused on the correlation between competitive sports and temporomandibular dysfunction. Their limitations can be summarized as follows: different sample sizes and different analyzed TMD signs and/or symptoms, thus allowing only limited comparability. In addition, some parameters, such as gender and age, have not always been considered.

Persson et al. evaluated the prevalence of TMD symptoms in wrestlers and non-wrestlers (26 males each) using a questionnaire and a clinical examination, but an overall prevalence was not stated. The most frequent symptoms identified in the questionnaire were crepitation of the TMJ in the wrestler group and headache, as well as crepitation in the control group [17].

Weiler et al. compared male basketball players versus a CG of non-athletes (46 males and 41 males, respectively) and female basketball and handball players versus a CG of non-athletes (89 females and 72 females, respectively) in two different studies, but with the same study design, using a questionnaire as screening method [11,15]. If at least one of the findings was positive in the questionnaire, an additional functional analysis was carried out. No significant differences were found between the groups in both studies. Tenderness on palpation of the masticatory muscles was the predominant symptom in all groups.

Zamora-Olave et al., in two different studies, reported a prevalence of TMD of 11.7% in field hockey players (38/325 subjects of both sexes) and a prevalence of 20.2% in water polo players (70/347 subjects of both sexes), through a questionnaire, without information on the frequency of individual symptoms [18].

Using the clinical Helkimo index, Mendoza-Puente et al. detected a TMD frequency in athletes (14 out of 18 male boxers and 9 out of 20 male handball players) of 60.53% [39], while Bonotto et al., using the RDC/TMD, diagnosed Axis I temporomandibular dysfunctions in 54.2% of competitive karatekas (13/24 subjects of both sexes), in 17.6% of amateur karatekas (3/17 subjects of both sexes), in 61.5% of competitive mixed martial arts athletes (8/13 subjects of both sexes) and 14.3% of non-athletes (4/28 subjects of both sexes) [16]. The most common symptom in all four groups was a displacement of the disk, while tenderness on palpation of the masticatory muscles was prevalent in the group of non-athletes.

Gay-Escoda et al. found bruxism as the most common sign (30% or 9/30 of male subjects) of temporomandibular dysfunction in professional soccer players [40].

Recently Freiwald et al. published a literature review based on this topic, taking into account nine studies [29]. TMD frequency resulted between 11,7% and 100% for athletes and 11,11% and 14,3% for non-athletes. These data suggest a general trend: professional athletes suffer from temporomandibular dysfunctions more frequently than non-athletes and tend to show aggravated symptoms. This would mean that competitive athletes are exposed to greater stress, due to high training intensity, or psychological pressure caused by the increased training effort and competitions. Moreover, competitive athletes in a contact sport can develop temporomandibular dysfunction due to traumatic injuries in the orofacial area. This can explain, for example, the high values for boxers (77.77%) [39] or competitive MMA athletes (61.5%) [16].

According to Bueno et al. [9], the female gender is a significant risk factor for the development of TMD. Therefore, the present study examined exclusively male subjects to avoid a gender distribution bias. In addition, age is a crucial factor in the prevalence of TMD, with a peak in young adults up to mid-age [3,8]. For this reason, all examined groups of the present study were selected to have a mean age approximately between 22 and 25 years old, which coincides with the age range that is most engaged in competitive sports. Finally, the case numbers differ greatly between studies. Hence, in the present study, a comparison of same-sized samples from different contact sports was performed against a single control group matched for sex and age. It also seemed appropriate to adopt established, standardized methods such as the RDC or DC/TMD, which are approved for research purposes [29,35].

To define them as competitive, athletes had to be registered with a professional sports club and train no less than three times a week or no less than 8 h a week. In contrast, the subjects enrolled in the CG had to play an amateur sport or attend the gym no more than once a week.

There was no higher prevalence of comorbidity and drug or supplement assumptions in the sport groups than in the CG. Facial asymmetries and scars also did not appear more prevalent in athletes than in the controls, while the reported trauma, which could in turn predispose to the onset of TMD, resulted in being statistically significant for rugby, American football and basketball players compared to the CG, but not for the soccer players and boxers. Occlusal features were also evaluated. Except for crossbite in rugby athletes, no statistical differences were found between the athletes and the CG. Moreover, previous orthodontic treatments were significantly less prevalent in athletes than in the CG as well as midline deviations. The interaction between occlusion and TMD is still unclear, leading to controversial research conclusions. This is a consequence of the generally accepted multifactorial and multicausal character of TMD.

In the present work, findings are quite consistent towards a lack of clinically relevant association between TMD and dental occlusion [41,42].

Almost all rugby and American football athletes and all boxers reported wearing mouthguards, with a high statistical difference from the CG. No significant differences were found for the soccer players. Unexpectedly, no basketball player reported wearing a mouthguard, despite the high prevalence of trauma reported in this group. It is interesting to note at the same time that in the boxing group, where all athletes wear a mouthguard, the reported trauma, with episodes of acute temporomandibular disorder (TMD) pain, was not significant. Zamora-Olave described similar results for hockey players. One might think that, in addition to protecting the teeth from trauma, mouthguards may also have a protective role for the temporomandibular joints [43].

Regarding TMD symptoms, the prevalence was significantly higher in athletes than in the CG. In the history questionnaire, the rugby and American football groups had most symptoms of TMD with a significant difference from the CG: masticatory, neck and shoulder muscle pain, TMJ arthralgia for both groups and difficulty in opening the mouth for the rugby group rather than a headache in the temporal region for the American football group. The soccer group showed a significant difference to the CG for masticatory, neck and shoulder muscle pain. In the basketball players, only masticatory muscle pain was statistically significant. No differences were found for joint locks for each sports group. Surprisingly, the boxing group complained of no significant TMD symptoms compared to the CG except for tinnitus, which was significantly less frequent than in the CG. Except for the boxers, these findings confirm the general trend reported in the literature for a prevalence of TMD symptoms, especially the muscular ones, in competitive athletes compared to the general population.

On clinical examination, a statistical difference was found regarding myofascial pain for the soccer and American football groups compared to the CG for anterior temporal muscle pain. No statistical differences were found for the boxing, rugby and basketball groups, although this muscle was more painful for the latter two groups than the CG. No statistical differences were found between all groups of athletes and the CG for medial temporalis, posterior temporalis and trapezius pain. The sternocleidomastoid was painful in almost all members of all groups, including the CG, except for the boxing group. In these sportsmen, sternocleidomastoid, superficial masseter, medial pterygoid and lateral pterygoid were significantly less painful than in the CG. The digastric muscle was painful in the CG as well as in the American football, rugby and boxing groups. This muscle was significantly less painful for soccer and basketball players than in the CG.

Overall, myogenous TMD is the most common type in clinical settings, characterized by regional or localized pain of the masticatory muscles, including increased muscle tenderness, pain sensitivity and fatigue. This means that if palpation of the masticatory muscle causes pain only at the site of palpation, it is classified as MYA (local pain), while if it refers to structures outside of the muscle, it is classified as MFP (regional pain).

In the present study, the absence of comorbidities seems to reduce the risk of developing TMD and helps avoid pain persistence and treatment failure. About myofascial pain, a general trend can be identified, with a slight prevalence for athletes compared to the CG, with the exception of boxers [44].

Regarding TMD signs, no statistical differences among groups were found for opening, clicking and snapping. Closing click was statistically more significant for all groups of athletes than the CG, while crepitation resulted significant only for the rugby and American football groups.

There was no significant prevalence of bruxism, teeth clenching and wear facets in the athletes’ groups compared to the control one. At the same time, the indentation of lateral edges of the tongue was significantly lower in the basketball players than in the CG as well as the buccal occlusal line being significantly lower for the boxers and basketball players than in the CG.

People undergoing regular physical activity with a high consumption of sports drinks are most affected by tooth erosion. The exposition to the acid drinks’ intake could cause a reduction in the salivary pH, with dissolution of the calcium ions constituting the enamel and the dentine. In the present work, however, no correspondence was found between sport and dental erosion [45,46].

Regarding the mandibular kinematics, no statistical differences were found between the sports groups and CG in the measurement of opening, protrusion and left laterotrusion movements, except for the rugby players, who showed more limited movements in laterotrusion, with a statistical difference compared to soccer players and boxers. A statistical difference was also reported for endfeel measurements in rugby players versus controls, basketball and American football groups.

Finally, regarding the alterations of the mandibular opening, no statistical differences were found among groups for deflection, while the deviation resulted in being statistically significant for soccer, rugby and American football players. As well as TMD symptoms, TMD signs were more frequent in athletes with respect to the CG, above all for rugby and American football players.

About the recruitment of the CG, people referring to the hospital may complain of oral disturbances more frequently than global population, so this could be a point of weakness of the present study.

Another limitation lies in the decision of the Ethics Committee to allow only a clinical-observational study, prohibiting I and II level radiological investigations. This clinical approach implies a less accurate diagnosis regarding the presence/absence of condylar alterations, such as arthritis or arthrosis.

## 6. Conclusions

The data collected from this observational study seem to support a relationship between the prevalence of TMD symptoms and signs in competitive athletes in contact sports, especially in RG and AFG, compared to a control group of non-athletes. Instrumental tests, such as electromyography, could be used hereafter to evaluate the bioelectrical activity of the chewing muscles, in order to determine the relationship between these muscles and sports disciplines [47,48].

## Figures and Tables

**Table 1:** Mean ages (±SD) and number of samples per group.

Groups	Mean Age	±SD
SoG	22.7	2.78
RG	25.3	7.71
AFG	22.4	4.75
BoG	23.85	6.67
BaG	23.35	6.67
CG	22	3.09

**Table 2:** Comorbidity and drugs: comparison between different sports and controls.

Comorbidity and Drugs	SoG	RG	AFG	BoG	BaG	CG
Systemic Pathologies	^‡^ 0 (0.0%) ***	^‡^ 0 (0.0%) ***	^¥^ 3 (15%)	^¥^ 9 (45%)	^¥^ 6 (30%)	7 (35%)
Medications	^‡^ 0 (0.0%) *	^‡^ 0 (0.0%) *	^‡^ 0 (0.0%) *	^‡^ 2 (10%)	^‡^ 1 (5%)	5 (25%)
Supplements	^¥^ 6 (30%)	^¥^ 6 (30%)	^‡^ 2 (10%)	^¥^ 11 (55%)	^¥^ 6 (30%)	6 (30%)
Facial asymmetries	^‡^ 8 (40%)	^¥^ 13 (65%)	^¥^ 11 (55%)	^¥^ 10 (50%)	^¥^ 11 (55%)	12 (60%)
Reported Trauma	^¥^ 8 (40%)	^¥^ 18 (90%) *	^‡^ 19 (95%) ***	^‡^ 10 (50%)	^‡^ 19 (95%) ***	12 (60%)
Scars	^¥^ 8 (40%)	^¥^ 12 (60%)	^¥^ 4 (20%) *	^¥^ 10 (50%)	^¥^ 10 (50%)	11 (55%)

* 0.02 < *p* < 0.05, ** 0.01 < *p* < 0.02, *** *p* < 0.01; ^‡^ Fisher, ^¥^ Chi-square.

**Table 3:** Occlusion: comparison between different sports and controls.

Occlusion	SoG	RG	AFG	BoG	BaG	CG
Cross Bite	^¥^ 0 (0.0%)	^‡^ 7 (35%) *	^‡^ 5 (25%)	^‡^ 4 (20%)	^‡^ 5 (25%)	1 (5%)
Brodie	0 (0.0%)	0 (0.0%)	0 (0.0%)	0 (0.0%)	^‡^ 1 (5%)	0 (0.0%)
Open Bite	0 (0.0%)	0 (0.0%)	0 (0.0%)	^‡^ 1 (5%)	^‡^ 1 (5%)	0 (0.0%)
Deep Bite	^¥^ 6 (30%)	^¥^ 6 (30%)	^¥^ 9 (45%)	^¥^ 7 (35%)	^¥^ 7 (35%)	8 (40%)
**Orthodontic Treatments**	^¥^ 4 (20%) ***	^¥^ 4 (20%) ***	^¥^ 5 (25%) ***	^¥^ 9 (45%)	^¥^ 12 (60%)	14 (70%)
Presence of Protheses	^‡^ 3 (15%)	^‡^ 3 (15%)	^‡^ 1 (5%)	^‡^ 4 (20%)	^‡^ 3 (15%)	1 (5%)
Mouthguards	^‡^ 3 (15%)	^¥^ 16 (80%) ***	^¥^ 19 (95%) ***	^¥^ 20 (100%) ***	^‡^ 0 (0.0%) *	5 (25%)
Midline deviation	^¥^ 10 (50%)	^¥^ 11 (55%)	^¥^ 7 (35%) **	^¥^ 9 (45%)	^¥^ 7 (35%) **	15 (75%)

* 0.02 < *p* < 0.05, ** 0.01 < *p* < 0.02, *** *p* < 0.01; ^‡^ Fisher, **^¥^** Chi-square.

**Table 4:** TMD symptoms: comparison between different sports and controls.

TMD Symptoms	SoG	RG	AFG	BoG	BaG	CG
Masticatory Muscle Pain During Function	^‡^ 8 (40%) ***	^‡^ 8 (40%) ***	^¥^ 11 (55%) ***	^‡^ 2 (10%)	^‡^ 7 (35%) *	1 (5%)
Neck And Shoulder Muscles Pain	^¥^ 13 (65%) **	^¥^ 13 (65%) **	^¥^ 14 (70%) ***	^¥^ 8 (40%)	^¥^ 9 (45%)	5 (25%)
TMJ Arthralgia	^‡^ 4 (20%)	^¥^ 8 (40%) *	^¥^ 8 (40%) *	^‡^ 0 (0.0%)	^‡^ 4 (20%)	2 (10%)
Difficulty Opening Mouth	^‡^ 1 (5%)	^‡^ 5 (25%) *	^‡^ 1 (5%)	0 (0%)	^‡^ 3 (15%)	0 (0.0%)
Temple Headache	^¥^ 10 (50%)	^¥^ 10 (50%)	^¥^ 13 (65%) *	^¥^ 6 (30%)	^¥^ 5 (25%)	6 (30%)
Tinnitus	^¥^ 6 (30%)	^¥^ 9 (45%)	^¥^ 9 (45%)	^¥^ 4 (20%) *	^¥^ 7 (35%)	10 (50%)
Locks	^‡^ 3 (15%)	^‡^ 2 (10%)	^‡^ 1 (5%)	^‡^ 3 (15%)	^‡^ 0 (0.0%)	2 (10%)

* 0.02 < *p* < 0.05, ** 0.01 < *p* < 0.02, *** *p* < 0.01; ^‡^ Fisher, **^¥^** Chi-square.

**Table 5:** Muscular symptoms: comparison between different sports and controls.

Muscular Symptoms	SoG	RG	AFG	BoG	BaG	CG
Anterior Temporalis	^¥^ 9 (45%) **	^‡^ 7 (35%)	^¥^ 9 (45%) **	^‡^ 2 (10%)	^‡^ 5 (25%)	2 (10%)
Medial Temporalis	^‡^ 5 (25%)	^‡^ 6 (30%)	^‡^ 5 (25%)	^‡^ 2 (10%)	^‡^ 3 (15%)	3 (15%)
Posterior Temporalis	^‡^ 3 (15%)	^‡^ 6 (30%)	^‡^ 6 (30%)	^‡^ 0 (0.0%)	^‡^ 2 (10%)	2 (10%)
Sternocleidomastoid	^‡^ 19 (95%)	^‡^ 18 (90%)	^‡^ 16 (80%)	^¥^ 11 (55%) *	^¥^ 13 (65%)	17 (85%)
Trapezius	^¥^ 15 (75%)	^¥^ 9 (45%)	^¥^ 10 (50%)	^¥^ 9 (45%)	^¥^ 12 (60%)	14 (70%)
Digastric	^¥^ 2 (10%) ***	^¥^ 9 (45%)	^¥^ 10 (50%)	^¥^ 10 (50%)	^¥^ 5 (25%) *	12 (60%)
Superficial Masseter	^¥^ 15 (75%)	^¥^ 7 (35%)	^¥^ 11 (55%)	^¥^ 4 (20%) *	^¥^ 9 (45%)	10 (50%)
Medial Pterygoid	^¥^ 12 (60%)	^¥^ 15 (75%)	^¥^ 14 (70%)	^¥^ 3 (15%) **	^¥^ 5 (25%)	10 (50%)
Lateral Pterygoid	^‡^ 18 90%)	^¥^ 11 (55%)	^‡^ 20 (100%)	^¥^ 7 (35%) ***	^¥^ 9 (45%) *	16 (80%)

* 0.02 < *p* < 0.05, ** 0.01 < *p* < 0.02, *** *p* < 0.01; ^‡^ Fisher, ^¥^ Chi-square.

**Table 6:** Joint sound: comparison between different sports and controls.

Joint Sounds	SoG	RG	AFG	BoG	BaG	CG
Opening Click	^¥^ 12 (60%)	^¥^ 11 (55%)	^¥^ 13 (65%)	^¥^ 9 (45%)	^¥^ 9 (45%)	9 (45%)
Closing Click	^¥^ 12 (60%) ***	^¥^ 11 (55%) ***	^¥^ 12 (60%) ***	^‡^ 7 (35%) *	^¥^ 9 (45%) ***	**1 (5%)**
Snapping	^‡^ 4 (20%)	^‡^ 3 (15%)	^‡^ 4 (20%)	^‡^ 1 (5%)	^‡^ 3 (15%)	1 (5%)
Crepitation	^‡^ 3 (15%)	^‡^ 7 (35%) *	^‡^ 6 (30%) *	^‡^ 2 (10%)	^‡^ 4 (20%)	1 (5%)

* 0.02 < *p* < 0.05, ** 0.01 < *p* < 0.02, *** *p* < 0.01; ^‡^ Fisher, ^¥^ Chi-square.

**Table 7:** Parafunctional signs: comparison between different sports and controls.

Parafunctional Signs	SoG	RG	AFG	BoG	BaG	CG
Bruxism	^¥^ 5 (25%)	^¥^ 4 (20%)	^¥^ 4 (20%)	^¥^ 3 (15%)	^¥^ 4 (20%)	8 (40%)
Teeth Clenching	^¥^ 15 (75%)	^¥^ 16 (80%)	^¥^ 14 (70%)	^¥^ 8 (40%)	^¥^ 7 (35%)	11 (55%)
Wear Facets	^¥^ 7 (35%)	^¥^ 6 (30%)	^¥^ 5 (25%)	^¥^ 7 (35%)	^¥^ 7 (35%)	10 (50%)
Indentation of Lateral Edges of Tongue	^¥^ 12 (60%)	^¥^ 15 (75%)	^¥^ 13 (65%)	^¥^ 12 (60%)	^¥^ 6 (30%) **	14 (70%)
Buccal Occlusal Line	^‡^ 14 (70%)	^‡^ 15 (75%)	^¥^ 13 (65%)	^¥^ 8 (40%) ***	^¥^ 8 (40%) ***	17 (85%)

* 0.02 < *p* < 0.05, ** 0.01 < *p* < 0.02, *** *p* < 0.01; **^‡^** Fisher, **^¥^** Chi-square.

**Table 8:** Opening alteration: comparison between different sports and controls.

Opening Alteration	SoG	RG	AFG	BoG	BaG	CG
Deviation	^¥^ 10 (50%) *	^¥^ 11 (55%) *	^¥^ 11 (55%) *	^‡^ 3 (15%)	8 (40%)	4 (20%)
Deflection	^‡^ 3 (15%)	^‡^ 4 (20%)	^‡^ 5 (25%)	^‡^ 3 (15%)	5 (25%)	3 (15%)

* 0.02 < *p* < 0.05, ** 0.01 < *p* < 0.02, *** *p* < 0.01; **^‡^** Fisher, **^¥^** Chi-square.

**Table 9:** Mean values and ± standard deviation (± SD) of opening and endfeel measured in the Basketball group (BaG), Soccer group (SoG), American Football group (AFG), Boxing group (BoG), Rugby group (RG) and Control group (CG). Ordinary Anova and Tukey’s multiple comparisons tests were performed among the groups evaluated.

Groups		Opening (cm)		Endfeel (mm)	
		Mean	±SD	Mean	±SD
**A**	SoG	4.85	0.5	5.29	0.51
**B**	RG	4.85	0.46	4.85	0.72
**C**	AFG	5.05	0.6	5.43	0.52
**D**	BoG	5.08	0.64	5.32	0.69
**E**	BaG	5.215	0.53	5.58	0.62
**F**	CG	5.175	0.46	5.43	0.48
**Tukey’s multiple comparisons test**		**n.s.**	E vs. B ***; C vs. B * B vs. F *		

* 0.02 < *p* < 0.05, ** 0.01 < *p* < 0.02, *** *p* < 0.01.

**Table 10:** Mean values and ± standard deviation (SD) of right and left laterotrusion and protrusion measured in Basketball group (BaG), Soccer group (SoG), American Football group (AFG), Boxing group (BoG), Rugby group (RG) and Control group (CG). Ordinary Anova and Tukey’s multiple comparisons tests were performed among the groups evaluated.

Groups		R-Laterotrusion (mm)		L-Laterotrusion (mm)		Protrusion (mm)	
		Mean	±SD	Mean	±SD	Mean	±SD
**A**	SoG	9.75	2.66	9.70	3.18	6.15	1.96
**B**	RG	6.40	3.93	8.20	3.72	7.45	2.29
**C**	AFG	8.20	3.23	9.35	2.73	7.25	2.57
**D**	BoG	10.0	0.64	9.55	2.80	6.15	2.43
**E**	BaG	8.80	2.94	9.80	3.04	7.60	2.62
**F**	CG	10.65	3.88	8.00	2.70	7.25	2.17
**Tukey’s multiple comparisons test**		B vs. A**; B vs. D***; F vs. B***		**n.s.**		**n.s.**	

* 0.02 < *p* < 0.05, ** 0.01 < *p* < 0.02, *** *p* < 0.01.

## Data Availability

The data presented in this study are available on request from the corresponding author upon reasonable request.

## Supplementary Materials

The following supporting information can be downloaded at: https://www.mdpi.com/article/10.3390/dj10100180/s1, Supplementary File S1: questionnaire.
