# Supplementary material for: Effects of Contact Sports on Temporomandibular Disorders: An Observational Study
Source: Dent J (Basel). 2022 Sep 27;10(10):180. doi: 10.3390/dj10100180 (PMC9600988; doi:10.3390/dj10100180)
Supplement: Supplementary file 1 [file dentistry-10-00180-s001.zip › dentistry-1907051-supplementary.pdf]

UNIVERSITÀ DEGLI STUDI DI BARI

"ALDO MORO"

AZIENDA OSPEDALIERO-UNIVERSITARIA CONSORZIALE

POLICLINICO DI BARI

U. O. C. di ODONTOIATRIA

CARTELLA GNATOLOGICA

CARTELLA N°___________-CASO N°_______/20____

Cognome e Nome ______________________________ Sesso (M □ \ F □) Età ____aa

Nato\a il ___________ a _________________________ prov (___)

Residente a _________________________ in via\piazza ________________________

Prov (___) Nazione _________________ cap _________ tel______________________

Stato civile ____________________________ Figli ______________________________

Professione ____________________________

DIAGNOSI ____

DATA INIZIO CURA ______________ DATA FINE CURA _______________

MOTIVAZIONE (riportare testualmente le parole dette dal paziente):

……………………………………………………………………………………………
……………………………………………………………………………………………
……………………………………………………………………………………………

ANAMNESI PATOLOGICA REMOTA GENERALE

- ☐ Infezioni in atto: ……………………………………………………………………………
- ☐ Infezioni pregresse: ………………………………………………………………………...
- ☐ Ipertensione: ………………………………………………………………………………..
- ☐ Cardiopatie: ...……………………………………………………………………………
- ☐ Malattie vascolari: ………………………………………………………………………….
- ☐ Emopatie: …………………………………………………………………………………...
- ☐ Allergie: …………………………………………………………………………………….
- ☐ Immunopatie: ……………………………………………………………………………….
- ☐ Malattie reumatiche: ..……………………………………………………………………
- ☐ Malattie del collageno: ……………………………………………………………………..
- ☐ Malattie dermatologiche: …………………………………………………………………...
- ☐ Malattie ortopediche: ……………………………………………………………………….
- ☐ Malattie neurologiche: ……………………………………………………………………...
- ☐ Vertigini: ……………………………………………………………………………………
- ☐ Malattie ORL: ………………………………………………………………………………
- ☐ Malattie oculari: …………………………………………………………………………….
- ☐ Apparato G. I.: ……………………………………………………………………………...
- ☐ Apparato urologico: ………………………………………………………………………...
- ☐ Sistema endocrino: …………………………………………………………………………
- ☐ Interventi chirurgici: ……………………………………………………………………….
- ☐ Incidenti e traumi: ………………………………………………………………………….
- ☐ Ansia\depressione:………………………………………………………………………….
- ☐ Fumo \ alcool \ droghe.
- ☐ Altro:
  ……………………………………………………………………………………….....
  …………………………………………………………………………………………

ANAMNESI FARMACOLOGICA

………………………………………………………………………………………………
………………………………………………………………………………………………
………………………………………………………………………………………………
………………………………………………………………………………………………
………………………………………………………………………………………………
………………………………………………………………………………………………
………………………………………………………………………………………………
………………………………………………………………………………………………

ANAMNESI PATOLOGICA REMOTA SPECIALISTICA

HA SUBITO:

- ☐ TERAPIE ODONTOIATRICHE:…………………………………………………………..
- ☐ TERAPIE ORTODONTICHE:……………………………………………………………..
- ☐ TERAPIE PROTESICHE:……………………………………………………………….
- ☐ CHIRURGIA ODONTOIATRICA:…………………………………………………………..

IN PASSATO E' STATO GIA'

- ☐ VISITATO PER QUESTO PROBLEMA? ………………………………………………..
- ☐ TRATTATO PER QUESTO PROBLEMA? (specificare tipi di apparecchiatura o farmaci utilizzati: ....
  ....

ANAMNESI PATOLOGICA PROSSIMA

- ☐ CEFALEA
- ☐ AFFATICAMENTO MUSCOLARE
- ☐ DISTURBI UDITIVI
- ☐ DOLORE IN FASE DI APERURA BUCCALE
- ☐ RUMORE IN FASE DI APERTURA BUCCALE:
  ☐ CLICK ☐ CLICK RECIPROCO ☐ SCHIOCCO ☐ CREPITIO
- ☐ DIFFICOLTA' A MASTICARE
- ☐ DOLORE DURANTE LA MASTICAZIONE
- ☐ RUMORI DURANTE LA MASTICAZIONE
- ☐ DIFFICOLTA' ALLA DEGLUTIZIONE

	INSORGENZA					
	NO	SI	NOTTE	GIORNO	RIPOSO	ATTIVITA'
CERVICALGIA						
BRACHIALGIA						
DORSALGIA						
LOMBALGIA						
SCIATALGIA						
PARESTESIA						

DA QUANDO (il sintomo più insopportabile):

<15 giorni	<3 mesi	>3 mesi	>6 mesi	______ anni

LOCALIZZAZIONE REGIONALE

	INSORGENZA						
	DX	SX	BIL	NOTTE	GIORNO	RIPOSO	ATTIVITA'
TESTA							
COLLO							
FACCIA							
ORECCHIO							
FRONTE							
NASO							
OCCHIO							

DISTRETTO CEFALICO				
CEFALEA				EMICRANIA
TEMPORALE	FRONTALE	AL VERTICE	OCCIPITALE	

TIPO (del sintomo più insopportabile): ☐ Acuto ☐ Gravativo ☐ Costrittivo

LIVELLO DEL DOLORE (VAS)

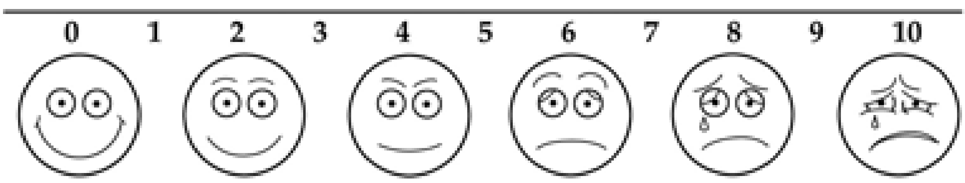

- FREQUENZA:
  ☐ GIORNALIERA ☐ SETTIMANALE ☐ >1 SETTIMANA ☐ MENSILE
- DURATA:
  ☐ COSTANTE ☐ SPORADICA ☐ INTERMITTENTE
- CAUSE CHE MODIFICANO IL DOLORE:
  ☐ IN PIEDI ☐ CAMMINANDO ☐ SUPINO ☐MANGIANDO ☐ ALTRO
  ……………………………………………………...……

QUANTO IL SUO PROBLEMA INTERFERISCE SULLA SUA VITA QUOTIDIANA?

0% 50% 100%

Qual è la sua opinione personale riguardo ai motivi che scatenano il dolore?
……………………………………………………………………………………………
……………………………………………………………………………………………

Ci sono elementi che disturbano il sonno?
……………………………………………………………………………………………
……………………………………………………………………………………………

Che cosa si aspetta dal trattamento?
……………………………………………………………………………………………
……………………………………………………………………………………………

Ci sono altri dati che non le sono stati richiesti e che ritiene importanti?
……………………………………………………………………………………………
……………………………………………………………………………………………

ESAME OBIETTIVO EXTRAORALE

TIPO FACCIALE		
MESIOFACCIALE	DOLICOFACCIALE	BRACHIFACCIALE

PROFILO DEI TESSUTI MOLLI		
NORMALE	CONVESSO	CONCAVO

ASIMMETRIE FACCIALI MACROSCOPICHE		
ASSENTE	DEV. DESTRA	DEV. SINISTRA

LABBRA	
COMPETENTI	INCOMPETENTI

CICATRICI	
PRESENTI<br>sede_______________________________________	ASSENTI

ESAME OBIETTIVO INTRAORALE

TESSUTI MOLLI	
IRREGOLARITA' DEI MARGINI LINGUALI	LINEA ALBA OCCLUSALE

MOLARI		
1ª CLASSE	Dx	Sx
2ª CLASSE	Dx	Sx
3ª CLASSE	Dx	Sx

CANINI		
1a CLASSE	Dx	Sx
2a CLASSE	Dx	Sx
3a CLASSE	Dx	Sx

INCISIVI	
1ª DIVISIONE	2ª DIVISIONE

OVERBITE: ________________mm   OVERJET ________________mm

CURVA DI SPEE			
NORMALE	PIATTA	PROFONDA	INVERSA

STATO PARODONTALE	
BUONO	INSUFFICIENTE

	DX	SX	BIL	TOT	PAR
OPEN LATERALE					
CROSS BITE					
BRODIE					
ESTRAZIONI					
MIGRAZIONI					
INCLINAZIONI					
ROTAZIONI					
AGENESIE					
SOPRANNUMERARI					
INCLUSIONI					
CARIE					
FACCETTE DI USURA					
OTTURAZIONI					
PROTESI FISSA					
PROTESI MOBILE					
AFFOLLAMENTO					
DIASTEMI					

CONTATTO DENTALE ANTERIORE IN C. O.

TESSUTALE	
1/3 CERVICALE	
1/3 MEDIO	
1/3 INCISALE	
CROSS BITE	

TIPO DI OCCLUSIONE

MUTUALMENTE PROTETTA			
CONTATTO PIUMA DEI FRONTALI			
	PT	LD	LS
IPERBILANCIATA			
BILATERALMENTE BILANCIATA			
FUNZIONE DI GRUPPO			
DISCLUSIONE DI GRUPPO			
DISCLUSIONE FRONTALE			

LOCALIZZAZIONE DI CONTATTI PREMATURI							
M	PM	C	I	I	C	PM	M
M	PM	C	I	I	C	PM	M

ESAME FUNZIONALE CLINICO

ISPEZIONE GENERALE (POSTURA)		
DEBOLE	SUFFICIENTE	BUONA

POSIZIONE DI RIPOSO DURANTE L'ESAME	CAPO	SIMMETRICA	ASIMMETRICA
	CORPO	SIMMETRICA	ASIMMETRICA

	ATTIVI	DOLORE		PASSIVI	DOLORE		ENDFEEL	
	mm	DX	SX	mm	DX	SX	positivo	negativo
APERTURA MASSIMA								
LATEROTRUSIONE SX								
LATEROTRUSION DX								
PROTRUSIONE DEVIAZ								
RETRUSIONE								

DEVIAZIONE DEL TRAGITTO DI APERTURA E CHIUSURA

(far compiere al paziente la massima apertura possibile)

			a quanti mm avviene
APERTURA		Sx	Mm
CHIUSURA		Sx	Mm
RITORNO IN ASSE DURANTE L'APERTURA			SI

CLICKING

APERTURA	Dx	Sx	CHIUSURA	Dx	Sx
INIZIO			FINE		
INTERMEDIO			INTERMEDIO		
FINE			INIZIO		
SCOMPARSA CON MANOVRA IN PROTRUSIVA					
CREPITIO					
LOCKING					

PARAFUNZIONI		
SERRAMENTO	DIGRIGNAMENTO	MORDERE ALTRO --

ABITUDINI VIZIATE				
SUCCHIAMENTO DEL POLLICE	INTERPOSIZIONE LABIALE	DEGLUTIZIONE ATIPICA	ONICOFAGIA	RESPIRAZIONE ORALE

PALPAZIONE MUSCOLARE (tensione e dolorabilità)

DX			TRIGGER POINT		SX			TRIGGER POINT
				TEMPORALE ANTERIORE				
				TEMPORALE MEDIO				
				TEMPORALE POSTERIORE				
				STERNOCLEIDOMASTOIDEO CAPO STERNALE				
				STERNOCLEIDOMASTOIDEO CAPO CLAVICOLARE				
				DIGATRICO VENTRE POSTERIORE				
				DIGASTRICO VENTRE ANTERIORE				
				BASE DEL CRANIO/PARTE POSTERIORE DEL COLLO				
				TRAPEZIO SUPERIORE				
				TRAPEZIO INFERIORE				
				MASSETERE SUPERFICIALE				
				MASSETERE PROFONDO				
				TEMPORALIS TENDON				
				PTERIGOIDEO ESTERNO CAPO SUP				
				PTERIGOIDEO ESTERNO CAPO INF				

ESAMI RICHIESTI

	DATA	DIAGNOSI
RX OPT	______________	
STRATIGRAFIA ATM	______________	
TELERADIOGRAFIA LATERO-LATERALE	______________	
TELERADIOGRAFIA POSTERO-ANTERIORE	______________	
TELERADIOGRAFIA ASSIALE	______________	
RMN ATM	______________	
ARCO FACCIALE	______________	
KINESIOGRAFIA	______________	
AXIOGRAFIA	______________	
ELETTROMIOGRAFIA	______________	
CEFALOMETRIA L-L	______________	
CEFALOMETRIA P-A	______________	
CEFALOMETRIA ASSIALE	______________	

CONSULENZE

	DATA	DIAGNOSI
CHIRURGICA	______________	
PROTESICA	______________	
OCULISTICA	______________	
ORL	______________	
NEUROLOGICA	______________	
FISIATRICA	______________	
PSICHIATRICA	______________	

DIAGNOSI

- ALTERAZIONI DI ORIGINE MUSCOLARE……………………………………………
- IPOMOBILITA'……………………………………………………………………….
- IPERMOBILITA' CONDILARE…………………………………………………..
- DISLOCAZIONE DEL DISCO: □INIZIALE
  □INTERMEDIA
  □FINALE
- ASIMMETRICA MORFOLOGICA………………………………………………..
- ASIMMETRIA FUNZIONALE………………………………………………………
- LOCKING……………………………………………………………………………..
- ARTROSI ATM……………………………………………………………………….
- ALTRO…………………………………………………………………………...

  ……………………………………………………………………………………

ANNOTAZIONI

………………………………………………………………………………………………
………………………………………………………………………………………………
………………………………………………………………………………………………
………………………………………………………………………………………………
………………………………………………………………………………………………
………………………………………………………………………………………………

TERAPIA CONSIGLIATA

FARMACOLOGICA

………………………………………………………………………………………………
………………………………………………………………………………………………
………………………………………………………………………………………………
………………………………………………………………………………………………
………………………………………………………………………………………………
………………………………………………………………………………………………

CON PLACCA

………………………………………………………………………………………………
………………………………………………………………………………………………
………………………………………………………………………………………………
………………………………………………………………………………………………
………………………………………………………………………………………………
…………………………………………………………………………………………...

OCCLUSALE

………………………………………………………………………………………………
………………………………………………………………………………………………
……………………………………………………………………………………………
………………………………………………………………………………………………
………………………………………………………………………………………………

DIARIO

DATA	VALUTAZIONI CLINICHE-	VAS
